# Burden of melanoma in China and its provinces from 1990 to 2021: an analysis for the Global Burden of Disease Study 2021

**DOI:** 10.3389/fpubh.2024.1486617

**Published:** 2024-12-09

**Authors:** Lingling Yu, Fanshu Yan, Jinlei Qi, Lijun Wang, Maigeng Zhou, Peng Yin

**Affiliations:** National Center for Chronic and Non-Communicable Diseases Control and Prevention, Chinese Center for Disease Control and Prevention, Beijing, China

**Keywords:** melanoma, burden of disease, mortality, incidence, public health

## Abstract

**Background:**

The incidence of melanoma in China has been increasing over the past few decades. This study aimed to investigate the burden of melanoma at both national and subnational level in China, where the population is rapidly aging.

**Methods:**

The annual melanoma data from 1990 to 2021 was collected from the Global Burden of Disease (GBD) 2021 China subnational study. Number of cases and age-standardized rates were estimated for incidence, mortality, prevalence, disability-adjusted life-years (DALYs) of melanoma by age and sex at subnational level with 33 province-level administrative units. Joinpoint regression model was used to evaluate the trends in disease burdens attributable to melanoma across time. A decomposition method was used to attribute changes in total deaths and DALYs to three explanatory components: population growth, population aging, and change of age-specific rates.

**Results:**

Over the past 30 years, the age-standardized incidence rate (ASIR) of melanoma in China has shown an upward trend. The ASIR of melanoma in 2021 was 0.7 per 100,000 (95%UI 0.4–0.9), representing an increase of 89.2% (95% UI: 14.7–157.9%) from 1990. Among younger adults aged <60 years, melanoma was more common in men, whereas among older adults who were aged >60 years, it was more common in women. The ASIR was higher in the coastal provinces in 2021 and the age-standardized rates (ASR) of DALYs was generally higher in the western provinces. Total numbers of death and DALYs of melanoma increased over the study period, mainly driven by population aging in China.

**Conclusion:**

China has experienced a substantial increase in the burden of melanoma from 1990 to 2021. It is beneficial to develop more targeted strategies for older adults populations, especially for women, to reduce the melanoma burden throughout China, particularly in some coastal and western provinces.

## Introduction

Melanoma is the most deadly skin cancer originating from melanocytes, and the prognosis of patients with advanced-stage melanoma differs widely between countries (1, 2). The morbidity of melanoma is found to increase worldwide in the past decades (3, 4). Melanoma of skin is more common in Caucasian population and the highest incidence of which was observed in Australasia, followed by North America in 2020 (5). In addition, people who are in older age groups have higher incidence rates of melanoma (6). In parallel, mortality rates have also continued increasing in the oldest patient groups (5). China is predicted to have one of the fastest growing aging populations in the world (7) and will face a heavy burden of melanoma in the future.

Only a few studies explored the burden of melanoma in Chinese population. Wu et al. (8) analyzed data from early round of Global Burden of Disease (GBD) 2017 and found that there has been a substantial increase in the burden of melanoma over the last decade in China. Bai et al. (9) investigated the long-term trends in the incidence and mortality of malignant melanoma, showing that age-standardized incidence rate (ASIR) of melanoma has increased from 1990 to 2019. With rapid population aging and economic development in recent years coupled with significant differences among provinces in China, existing studies may not accurately describe the current burden of melanoma across all provinces and there’s a need to comprehensively and multi-dimensionally investigate the burden of melanoma across different provinces using the latest research data. What’s more, little is known about the drivers of observed changes of burden caused by melanoma in China which could reflect the impact of three explanatory components including population growth, population aging and change of age-specific rates on melanoma burden.

Herein, we presented an updated analysis of the melanoma burden in China from 1990 to 2021 based on the most recent GBD 2021 China subnational study, and analyzed the incidence, mortality, prevalence and disability-adjusted life-years (DALYs) of melanoma by age and sex at both national and subnational level to make better targeted melanoma control policies. We also explored the potential contributions of three components which may drive the changes of death and DALYs due to melanoma during 1990–2021, in order to address corresponding interventions.

## Methods

### Overview

The method used in this study follows the general analytic framework used by GBD 2021 study (10, 11). In brief, the GBD 2021 study offers a comprehensive and up-to-date analysis of the global burden of 371 diseases and injuries for 204 countries and 811 subnational locations from 1990 to 2021. Additional information on GBD 2021 study can be accessed from the Global Health Data Exchange (GHDx) GBD 2021 website. In this study, number of cases and age-standardized rates from 1990 to 2019 were estimated for incidence, mortality, prevalence, DALYs of melanoma by age and sex in China and its 33 provincial level administrative units including 31 mainland provinces, autonomous regions and municipalities, and Hong Kong and Macau Special Administrative Regions.

### Data source

The mortality was estimated based on Disease Surveillance Points System, Maternal and Child Surveillance System, China Center for Disease Control and Prevention death reporting system, vital registration system for Hong Kong and Macao. Data sources for non-fatal outcomes were based on systematic reviews of scientific publications in China and the world. In this study, all ICD-10 codes (C43-C43.9, D03-D03.9, D22-D23.9, D48.5) and ICD-9 codes (172–172.9) of melanoma were included. All these datasets have been elaborated in previous studies (12). A full list of the data input resources used to estimate health outcomes from melanoma can be downloaded from the Sources Tool of GHDx.

### Measures

The GBD estimation process involves data collection and adjustments, estimation of all indicators by using specific modeling strategies, model validation and adjustment of results. Detailed descriptions have been reported in the GBD capstone publications (10, 11). Meta-regression-Bayesian, regularized, trimmed (MR-BRT) was used to adjust the uncertainty in the collected data and observations with greater uncertainty were given less weight in the model.

### Estimates of mortality and years of life lost

The Cause of Death Ensemble model (CODEm) developed specifically for GBD was used to estimate the mortality of melanoma. It estimated deaths by location, age, sex and year by combining the results through an ensemble of statistical models and systematically testing combinations of covariates for out-of-sample predictive validity (10). CODEm has a diverse pool of models to optimize algorithms, predictive validity tests while many other modeling strategies are also possible and it takes long time to process the data for each model (13). Years of life lost (YLLs) were calculated by multiplying deaths due to melanoma by the standard life expectancy at the age that death occurred (10).

### Estimates of prevalence, incidence and years lived with disability

Melanoma incidence and survival were used to estimate the 10-year prevalence. Total prevalence was distributed into four sequelae reflecting varying degrees of disability: (i) diagnosis and treatment, (ii) remission, (iii) metastatic and (iv) terminal. Spatiotemporal Gaussian process regression (ST-GPR) was the main methods for estimates of the prevalence over time, age, and location (10). ST-GPR is a set of regression methods that analyses heterogeneous and incomplete data requiring statistical smoothing while it may not capturing complex and high-non-stationary dynamics in spatiotemporal data perfectly and the complexity of model selection and hyperparameter optimization may increase with the amount of input data (14). Years lived with disability (YLDs) were calculated by multiplying the prevalent counts of sequelae by their respective disability weights, for melanoma. DALYs were calculated by summing YLDs and YLLs.

### Statistical analysis

Number of cases and age-standardized rates on incidence, deaths, prevalence, DALYs were used to fully describe the impact of melanoma on Chinese population. We analyzed the variation of melanoma by age group and sex at subnational level with 33 province-level administrative units, all of which were referred to as provinces in this study. The age group was defined for each 5-year from 15 to 20 years to 80 years and older. The rates were standardized according to the GBD world population and were reported per 100,000 person-years. The 95% uncertainty intervals (UIs) were produced for every metric using the 25th and 975th ordered 500 draw values of the posterior distribution and it was propagated at each step in the estimation process (10). Analyses were carried out with R (version 4.3.1). Joinpoint (version 5.0) was used to create the regression model to evaluate the trends in disease burdens attributable to melanoma across time. Default grid search method and permutation test was selected when setting the model. The minimum and maximum number of join points to fit were 0 and 6, respectively, according to our data points. We also calculated the average annual percentage change (AAPC) and the 95% confidence interval (95% CI) to describe the fluctuation trend. A two-sided *p-*value of less than 0.05 was considered statistically significant. ArcMap (version 10.7) was used to describe the regional distribution of melanoma in China.

A decomposition method developed by Gupta (15) was used to attribute changes in total numbers of death and DALYs to three explanatory components: population growth, population aging, and change of age-specific rates. We calculated the fraction of change in deaths and DALYs by cause from each component using counterfactual scenarios, changing the level of one factor from 1990 to 2021, with all other factors held constant (16). Take deaths for example, based on counterfactual theory, in scenario one, it was assumed that the number of population in the whole country in 2021 was the actual level, and the age structure and age-specific rates of death for melanoma were the same in 2021 as those in 1990, the expected number of deaths in 2021 was estimated by multiplying the number of population in 2021, age-specific mortality rate in 1990, and the age structure in 1990. In scenario two, the number of population and age structure was assumed to be as actual as in 2021, while the age-specific rates of death were held constant to 1990. During 1990–2021, the change attributed to population growth was then estimated by subtracting the actual deaths in 1990 from scenario one, the change attributed to population aging was calculated by determining the differences between scenario two and scenario one, and the change attributed to age-specific mortality rates was estimated by subtracting scenario two from the actual deaths in 2021 (17). One of the potential biases in Gupta’s decomposition analysis is that the selection of reference population needs to be considered when it comes to comparing different populations whereas this was not addressed in our study which only involves Chinese.

## Results

### Comparison of burden of melanoma in China and globally in 1990 and 2021

The number of newly diagnosed melanoma cases worldwide increased by 143.8% (95% UI: 132.1–153.1%) since 1990, while the incidence case in China has increased by 313.5% (95% UI: 147.6–472.7%) from 3,250 (95% UI: 2,093–4,086) to 13,437 (95% UI: 7,198–17,979) (Table 1). The ASIR of melanoma in China in 2021 was 0.7 per 100,000 (95% UI: 0.4–0.9), representing an increase of 89.2% (95% UI: 14.7–157.9%) from 1990 which was four times higher than the globe. The number of prevalence for melanoma in China increased by 1032.4% (95% UI: 574.5–1529.4%) during the study period, sixfold of the global increasing rate. The prevalence rates of melanoma in China increased by 518.4% (95% UI: 270.3–776.6%) from 1990 to 2021, while the global change is 32.6% (95% UI: 27.0–37.0%). Approximately 5,373 (95% UI: 2,849–7,106) persons died of melanoma in China in 2021 and the numbers of death increased by 109.0% (95% UI: 25.9–186.8%) as compared to 1990 (Table 1).

**Table 1:** The age-standardized rate, numbers and percent change for melanoma globally and for China, 1990–2021.

		Global			China		
		1990	2021	Change (%)	1990	2021	Change (%)
Incidence	Number	124,320 (119,604–127,610)	303,105 (281,718–318,905)	143.8 (132.1–153.1)	3,250 (2,093–4,086)	13,437 (7,198–17,979)	313.5 (147.6–472.7)
	Rate (per 100,000)	3.0 (2.9–3.1)	3.6 (3.3–3.7)	19.3 (13.6–23.6)	0.4 (0.2–0.5)	0.7 (0.4–0.9)	89.2 (14.7–157.9)
Prevalence	Number	833,216 (813,313–849,961)	2,177,566 (2,057,879–2,274,068)	161.3 (150.3–170.0)	7,172 (4,476–3,004)	81,219 (42,975–109,989)	1032.4 (574.5–1529.4)
	Rate (per 100,000)	19.1 (18.6–19.5)	25.4 (24.0–26.5)	32.6 (27.0–37.0)	0.7 (0.4–0.8)	4.2 (2.2–5.6)	518.4 (270.3–776.6)
Death	Number	33,061 (30,546–34,530)	61,550 (54,852–66,265)	86.2 (71.2–96.4)	2,571 (1,681–3,274)	5,373 (2,849–7,106)	109.0 (25.9–186.8)
	Rate (per 100,000)	0.8 (0.8–0.9)	0.7 (0.7–0.8)	−13.8 (−20.3–9.3)	0.3 (0.2–0.4)	0.3 (0.1–0.4)	−11.3 (−45.6–19.1)
DALYs	Number	1,045,778 (959,373–1,103,849)	1,678,836 (1,474,534–1,837,369)	60.5 (45.6–71.9)	88,682 (54,718–110,730)	153,206 (82,541–204,247)	72.8 (2.9–139.7)
	Rate (per 100,000)	24.3 (22.4–25.6)	19.6 (17.2–21.5)	−19.3 (−13.7–26.6)	9.0 (5.7–11.2)	7.8 (4.2–10.4)	−13.2 (−47.7–18.8)

Age-standardized rate (1/100,000) and data shown as rate (95% uncertainly interval). DALY, disability-adjusted life-year.

### Temporal trends of burden of melanoma from 1990 to 2021

**Figure 1:**
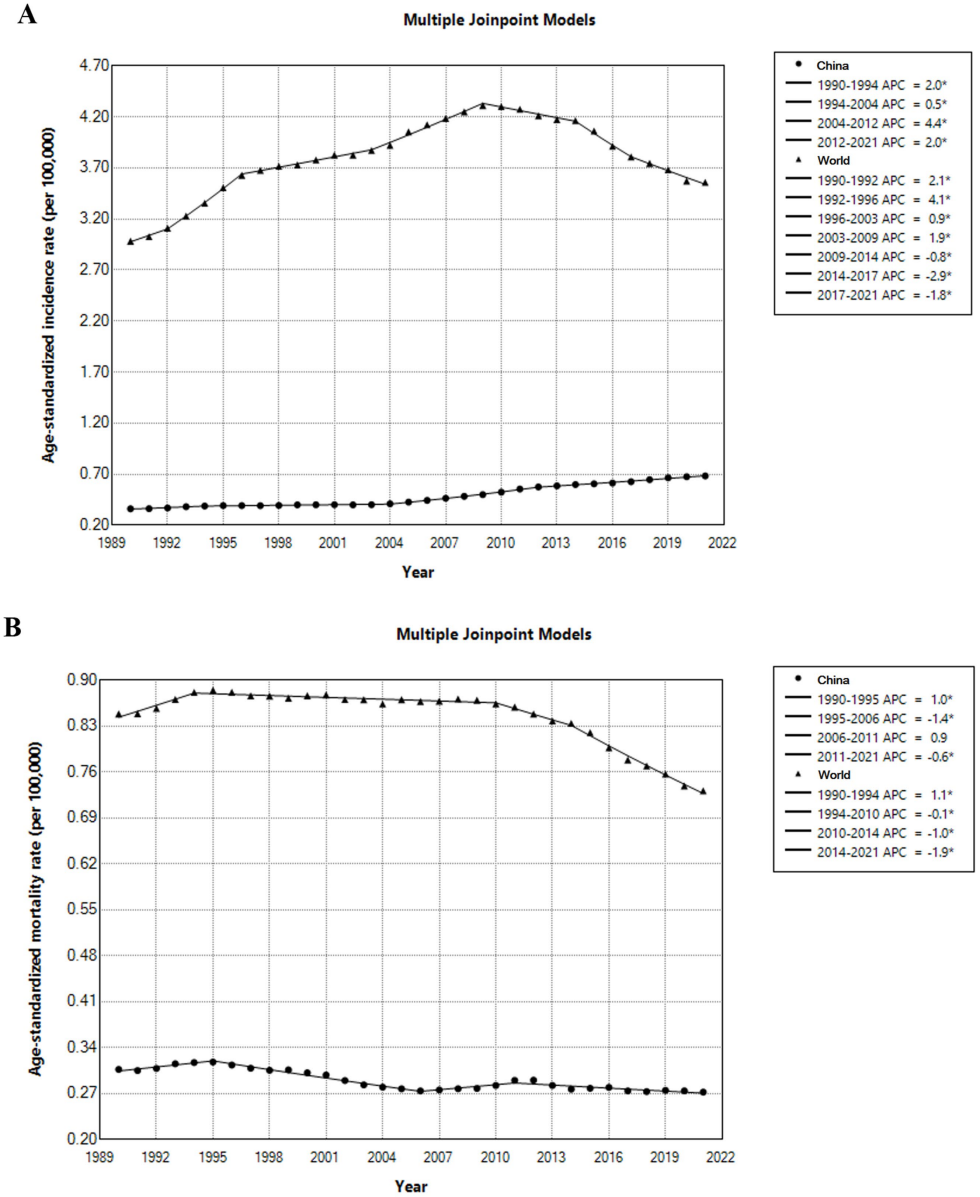
Temporal trends of incidence **(A)** and mortality **(B)** for melanoma globally and for China, 1990–2021.

China had lower ASIR and age-standardized mortality rate (ASMR) than global average over the past 30 years. The ASIR for melanoma in China had gone through four stages of significant increase from 1990 to 2021. In the period 2004–2012, the incidence rate showed fastest increase (4.4%). The ASMR in China decreased significantly during 1995–2006 by an average of 1.6% per year (Figure 1).

**Figure 2:**
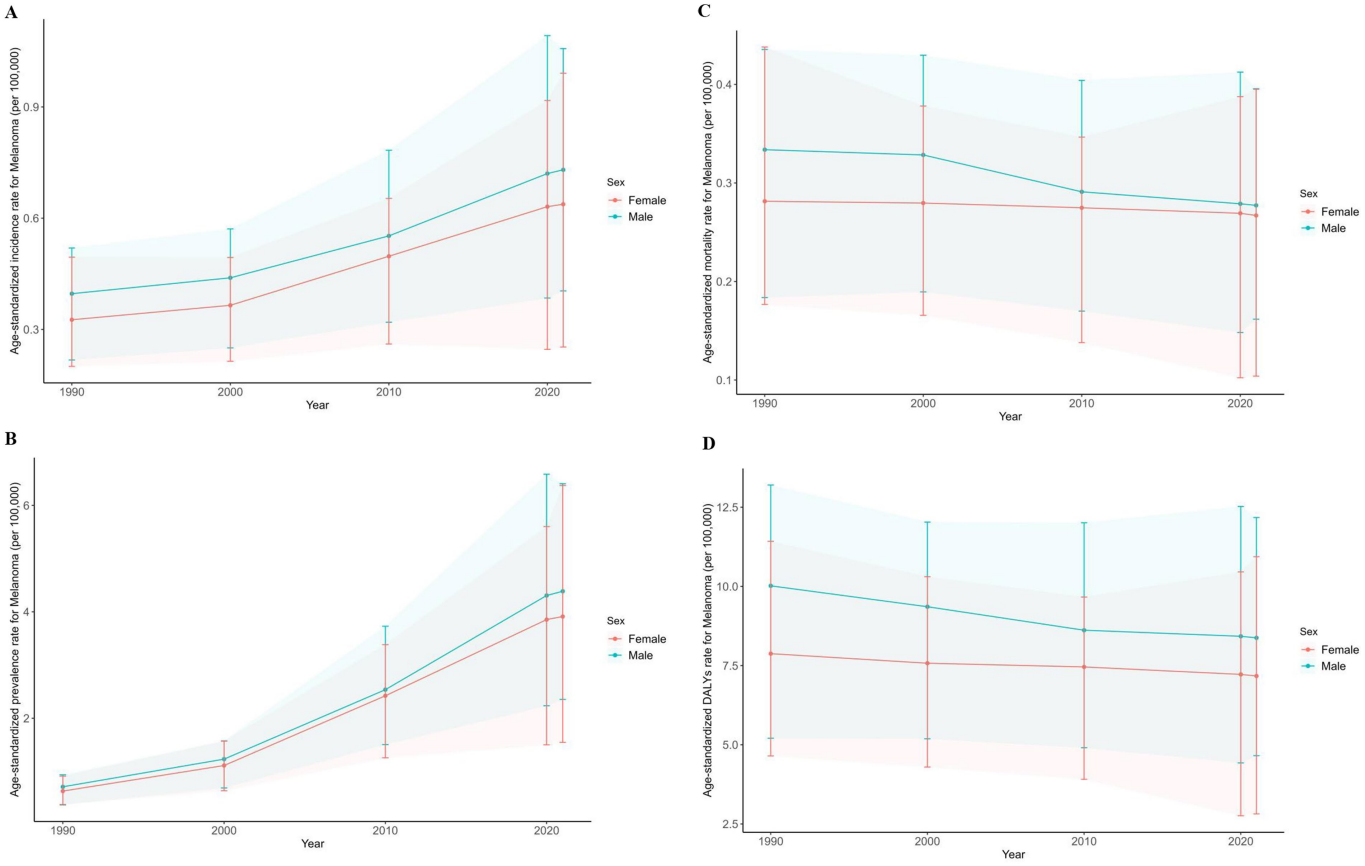
Age-standardized rate of incidence **(A)**, mortality **(B)**, prevalence **(C)** and DALYs **(D)** for melanoma in China from 1990 to 2021. DALY, disability-adjusted life-year.

The ASIR in both sexes had risen continuously from 1990 to 2021 and had a drastic rise from 2000 and the rate was consistently higher in men than in women. Between 1990 and 2021, the age-standardized rates (ASR) of prevalence in both sexes rose steadily (Figure 2). There’s a slight decreasing trend in ASMR and ASR of DALYs from 1990, and the decline was greater for men but still higher than women.

### Age and sex distribution pattern

**Figure 3:**
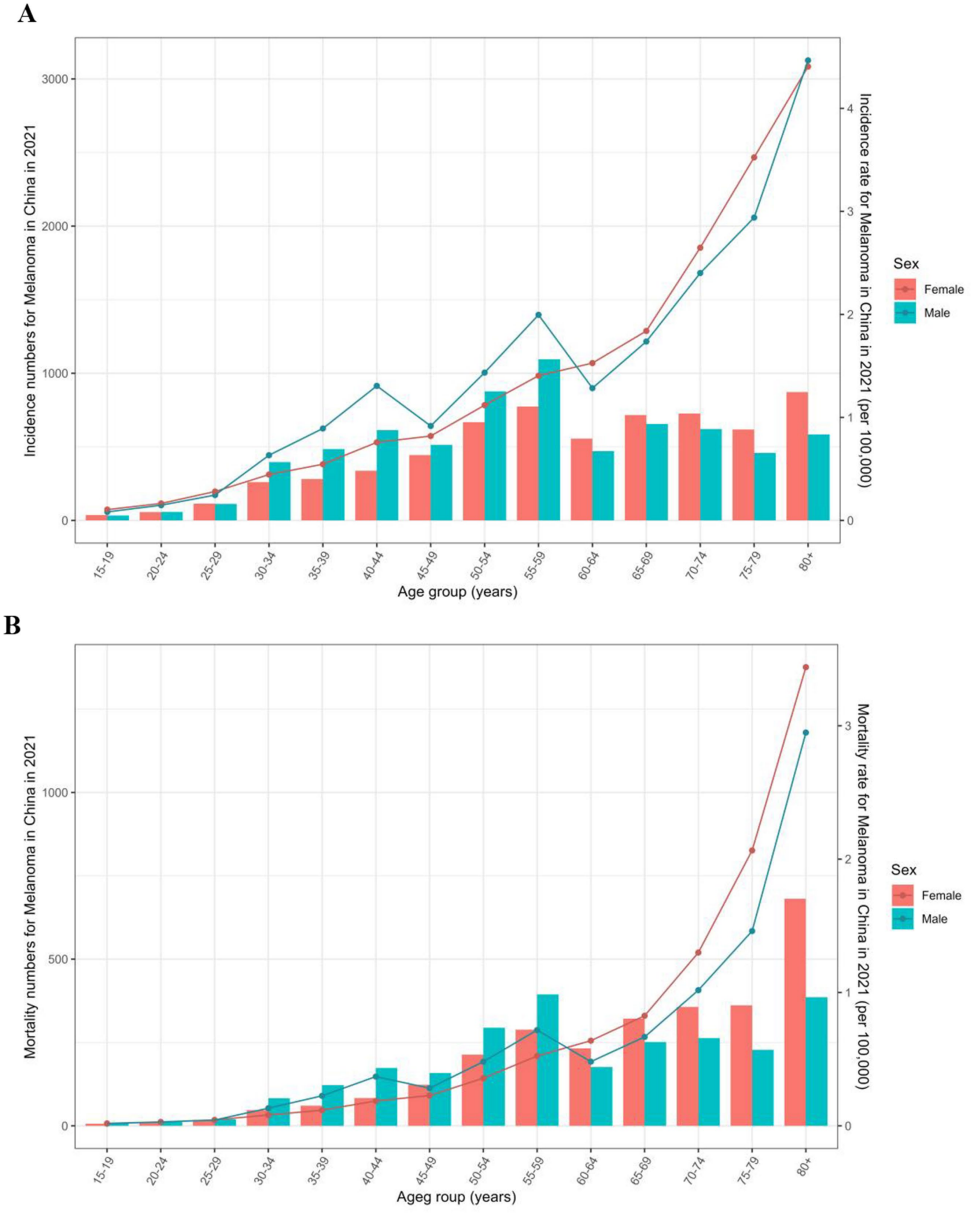
Incidence **(A)** and mortality **(B)** rate by sex and age for melanoma in China in 2021.

In 2021, men aged 55–59 years had the most incident cases and women over 80 years of age had the highest mortality rate compared to men and women in other age groups (Figure 3). Overall, the rate of incidence and mortality due to melanoma increased with age, and patients most affected by upward melanoma trends were those older than 60 years. Among people younger than 60 years, the incidence and mortality of melanoma was higher in men, but after age 60, the rate for women exceeds that of men.

### Geographic differences in China

**Figure 4:**
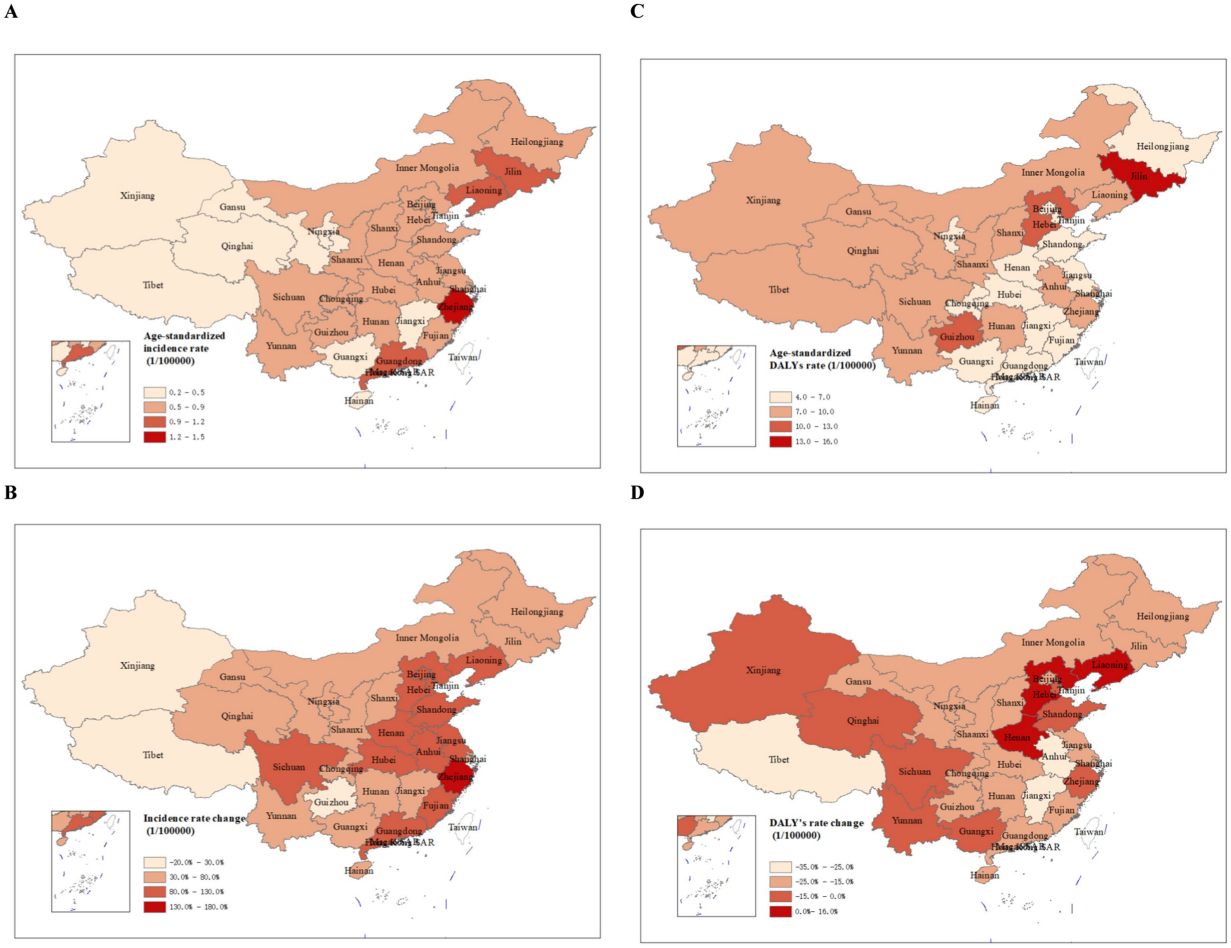
The age-standardized rate of incidence **(A)** and DALYs **(C)** in 2021 and percent change of incidence **(B)** and DALYs **(D)** from 1990 to 2021 in China. DALY, disability-adjusted life-year.

Significant geographic variations were observed for age-standardized incidence and DALYs rates of melanoma in China, 2021 (Figure 4). The ASIR was higher in the clustered eastern, northeast as well as coastal provinces (Figure 4A). The provinces with the highest ASIR per 100,000 in 2021 were Zhejiang (1.2, 95% UI: 0.5–1.7), Jilin (1.1, 95% UI: 0.3–1.8), Guangdong (1.0, 95% UI: 0.5–1.4) and Liaoning (0.9, 95% UI: 0.4–1.3) in the eastern and northeast China. Tibet had the lowest ASIR of melanoma (0.3, 95% UI: 0.2–0.4). Among 33 provinces, 32 showed an increasing trend in ASIR from 1990 to 2021, with the largest growth (174.8, 95% UI: 0.5–3.1; Figure 4B) seen in Zhejiang. The ASR of DALYs were generally higher in the western provinces and the greatest DALYs rates were in Jilin (13.7, 95% UI: 4.1–21.3), Hebei (11.7, 95% UI: 4.3–18.7) and Guizhou (11.4, 95% UI: 4.6–18.5; Figure 4C). Between 1990 and 2021, the ASR of DALYs decreased in 30 of 33 provinces (90.9%; Figure 4D).

### Decomposition of changes

Decomposition analysis of changes of deaths showed an overall 109.0% increase from 1990 to 2021, which were offset by the effect of changes of mortality rate (27.9%); while 115.9 and 20.9% of the changes in death case were attributable to population aging and population growth, respectively. Similar pattern was observed for both sexes (Table 2). The DALYs of melanoma increased over the study period mainly driven by population aging (79.9%).

**Table 2:** Percentage of contribution for changes of numbers of death and DALYs due to melanoma between 1990 and 2021 in China, by sex.

Cause	Gender	Population growth	Population aging	Change in age-specific rate	Total change
Deaths	Both	20.9%	115.9%	−27.9%	109.0%
	Male	20.0%	105.9%	−41.0%	84.8%
	Female	21.9%	126.0%	−10.5%	137.4%
DALYs	Both	20.9%	79.9%	−28.1%	72.8%
	Male	20.0%	72.2%	−33.8%	58.4%
	Female	21.9%	88.4%	−18.3%	92.1%

DALY, disability-adjusted life-year.

## Discussion

Based on the most recent epidemiological data from the GBD 2021 China subnational study, we found that although there was a slight decrease in ASMR and ASR of DALYs, the ASIR and ASR of prevalence in both sexes have risen continuously in the past three decades. Among younger adults aged <60 years, melanoma was more common in men, whereas among older adults who were aged >60 years, it was more common in women. The ASIR was higher in the coastal regions in China in 2021 and the ASR of DALYs was generally higher in the western provinces. Total numbers of death and DALYs of melanoma increased over the study period, mainly driven by population aging in China.

Over the past 30 years, the ASIR of melanoma in China has shown an upward trend, which may be due to increased radiation exposure, and people’s heightened awareness of skin lesions and improved diagnosis (5). Exposure to ultraviolet (UV) radiation in sunlight is one of the environmental risk factors for melanoma. Global warming also may result in increases in air pollutants, acid deposition, ozone depletion and exposure to UV radiation, increasing risk of melanoma (18). Improved public awareness of suspicious pigmented lesions and early, accurate detection has led to more melanoma cases being detected and treated in time. The upward prevalence has been influenced to some extent by the demographic changes in China. Population aging and growth has been rapid over the past few decades in China that it has caused a steady increase in the prevalence of disease (19). The ASMR and ASR of DALYs for cutaneous malignant melanoma in China have declined slightly, which may be due to the accessibility and affordability of health care and advances in medical technology (12).

Men diagnosed with melanoma have higher ASIR and ASMR in 2021. Extrapolated rationale for higher ASIR in men might be the behavior difference between men and women (20). Men in China are less likely to use sun protection and therefore get more exposure to UV rays (21). An online survey showed that only 21% of Chinese participants applied sunscreen, while male participants were less likely to use solar protection (22). Additionally, men are less sensitive to suspicious lesions and are more likely to delay in symptomatic presentation leading to advanced stage of disease than women (23). There are some evidence showing that men have significantly longer delay in seeking medical attention than women whose median and quartiles of delay time were longer than women, especially in some kinds of melanoma (21, 24).

Regarding the sex-age-specific incidence of melanoma, our study is not exactly consistent with the findings of previous studies that were conducted in fair-skinned populations (21, 25). Previous studies observed higher rates of melanoma in women before mid-life in the United States, Canada, Australia, and New Zealand and found an excess in men following mid-life (21, 26) while we found melanoma is more common in men under 60 years of age and women over 60 years of age in China. The reason may be due to differences in population behavior as well as ethnic variations (27). In general, adolescent and young adult women in fair-skinned populations have greater tanning risky behavior and socially determined aesthetic needs to the use of sunbeds, which causes higher melanoma incidence rates in their age (28). In our study, the higher incidence rate in young adult men may be, in part, due to more time spent outdoors, insufficient preventive behaviors and less likely to early detection (29). The higher incident rates observed in older women may be related to hormone change and specific sites of melanoma in Asian populations: acral lentiginous melanoma, a rare subtype of melanoma, is common in Asian, and the progressive decrease in estrogen with age loses its ability to regulate the growth and differentiation of tumor tissue, leaving the cancerous lesions more exposed in Chinese older female (27, 30). However, due to the long history of conflicting published results, whether menopause is associated with melanoma risk still needs to be resolved (30).

Regarding the spatial patterns of melanoma burden in China, the ASIR varied across provinces with higher ASIR in the eastern and northeast China, especially in some coastal provinces. Research conducted in other countries, such as Canada, and the United States has consistently revealed a similar pattern that melanoma is more common in coastal regions compared to inland areas (31–33). For example, it was discovered that Canadian maritime provinces, such as Prince Edward Island with a crude incidence rate of 33.9/100,000, and Nova Scotia with a rate of 30.8/100,000, had higher incidence rates of cutaneous melanoma compared to the inland provinces of Alberta (15.5/100,000), and Saskatchewan (14.5/100,000) (32). Within the same state, California, the incidence of melanoma in coastal counties was also significantly higher than in inland counties with an incidence rate ratio of 1.23 (95% CI: 1.05–1.43) (33).

We also found that the ASR of DALYs was generally higher in the western China. Differences in incidence and DALYs rates of melanoma in different regions of China may be explained by the disparity in medical care and UV exposure. In China, coastal provinces like Zhejiang are more economically developed than other regions and people have better access to better medical care (6, 34). The residents of this region may experience high incidence owing to the earlier detection and advanced medical technology (35). Conversely, the western region has a relatively weak economic foundation and a not-so-advanced health care leading to generally high DALYs rates. In addition, UV radiation as a major environmental risk factor for melanoma may also play a role in geographic disparities. The UV radiation depends on many factors like latitude, altitude, climate, anthropogenic behavior and so on (26). Some coastal and plain areas in China may be exposed to more frequent UV radiation. Most western provinces such as Guizhou are mostly mountainous areas with high altitudes where residents may also suffer high and intermittent UV exposure. The difference in melanoma between mountain and coastal residents may lies in the site of melanoma, with the former occurring more frequently on the head or neck and the latter on the trunk and extremities (31). However, our study did not investigate the difference between anatomical district of melanoma. More detailed analyses of body part of melanoma and geographical location will be needed to better understand its epidemiology. Anthropogenic behavior such as causing damage to the stratospheric ozone layer and using artificial light sources may also cause more UV exposure (36). With steel industry development, Jilin and Hebei (northeast China) may cause serious damage to the atmosphere, weakening absorption of UV rays. Thus people in these region may suffer more UV radiation and cause higher ASR of DALYs (36).

Population aging and growth are the main drivers of the increase in the numbers of death and DALYs of melanoma. Although China’s population growth is slowing, it is aging very rapidly and has the largest older adults population (37, 38). These demographic changes will likely exacerbate the burden on family and public healthcare systems (39). Therefore, effective preventive strategies and measures should be in place to reduce the increasing numbers of death and DALYs from melanoma. The Chinese Government attaches great importance to the prevention and control of cancer. While it is undoubtedly important to emphasize the urgency of control for leading causes of cancer types such as lung, stomach and liver cancer in China (40), tailored prevention and control measures for melanoma should also be in the agenda and steps should be taken to increase the awareness of this malignancy in Chinese population and reduce the burden in the context of an accelerating aging society.

We believe targeted primary prevention and education efforts could reduce the melanoma burden throughout China and high-incidence coastal and western provinces will benefit more (41, 42). Due to the low overall mortality of skin cancer and the insufficient evidence from randomized controlled trials to demonstrate the benefit of skin cancer screening in the general population (43), fewer countries have considered skin cancer screening in their statutory examinations. However, education and awareness about sun protection and the early symptoms of skin cancer remain appealing for this common and visible cancer, especially for melanoma, which could be eminently treated if detected early (44). In addition, patients and their surroundings should take the major responsibility for the early detection of melanoma (45). However, the typical early features of melanoma need to be clarified and publicized so that they can be promoted to population and primary care providers. If such preventive strategies could be implemented in China with a greater focus on specific populations or regions, it would be expected to increase the detection of early-stage melanoma in these rapidly expanding populations and reduce the burden of melanoma in the future.

Strengths of this study include the use of the most recent epidemiological data to show the spatial and temporal trends at both national and subnational level. Although the morbidity of melanoma in Chinese is not as high as western population, the incidence trend is still rising over the past three decades, which indicates the need of strengthening control and prevention of an often-neglected malignancy in the Chinese population. The current study also showed for the first time, the main drivers of the changes in total deaths and DALYs of melanoma using decomposition analysis from 1990 to 2021, providing important evidence to both clinicians and public health practitioners.

There are several limitations to our study. Firstly, the general limitations of the GBD data could affect our study. The study relies on GBD data, which may have fewer sources specific to melanoma in China, potentially affecting the precision of the estimates. However, the GBD modeling tool - Cause of Death Ensemble model, with its powerful modeling capabilities, could effectively compensate for the inadequacy of the data source (11). Secondly, we were not able to analyze the burden of histopathological subtypes of melanoma (like superficial spreading melanoma, nodular melanoma and lentigo maligna melanoma), as disease subtypes are not included in the GBD cause for now. Thirdly, underlying risk factors of melanoma, such as sunburns and the number of melanocytic nevi were not explored in this study due to data availability.

## Conclusion

From 1990 to 2021, China has experienced a substantial increase in the burden of melanoma. It is beneficial to develop more targeted strategies for older adults populations, especially for women, to reduce the melanoma burden throughout China, particularly some coastal and western provinces.

## Data Availability

The data of GBD 2021 used in this study can be accessed from the Global Health Data Exchange GBD 2021 website (https://vizhub.healthdata.org/gbd-results/ and https://ghdx.healthdata.org/gbd-2021).
